# Genetic Heterogeneity in Bartter Syndrome: Clinical and Practical Importance

**DOI:** 10.3389/fped.2022.908655

**Published:** 2022-06-03

**Authors:** Laura Florea, Lavinia Caba, Eusebiu Vlad Gorduza

**Affiliations:** ^1^Department of Nephrology-Internal Medicine, Faculty of Medicine, “Grigore T. Popa” University of Medicine and Pharmacy, Iasi, Romania; ^2^Department of Medical Genetics, Faculty of Medicine, “Grigore T. Popa” University of Medicine and Pharmacy, Iasi, Romania

**Keywords:** Bartter syndrome, tubulophathy, genetic heterogeneity, channel disease, rare disease

## Abstract

Bartter syndrome (BS) is a rare tubulopathy that causes polyuria, hypokalemia, hypochloremic metabolic alkalosis, and normotensive hyperreninemic hyperaldosteronism. It is characterized by locus, clinical, and allelic heterogeneity. Types 1–4 of BS are inherited according to an autosomal recessive pattern, while type 5, which is transient, is X linked. There are specific correlations between the clinical expression and the molecular defect, but since it is a rare disease, such studies are rare. Therapeutic interventions are different, being correlated with types of BS.

## Introduction

Bartter syndrome (BS) is a rare tubulopathy characterized by polyuria, hypokalemia, hypochloremic metabolic alkalosis, and normotensive hyperreninemic hyperaldosteronism (1). Incidence is 0.1/100,000 (2).

The main mechanism is the defective reabsorption of salt, mainly at the thick ascending limb (TAL) of the loop of Henle (3). TAL intervenes in the homeostasis of extracellular fluid by sodium reabsorption; homeostasis of calcium, magnesium, bicarbonate, and ammonium; and synthesis of uromodulin (Tamm-Horsfall protein) with a role in maintaining the composition of urinary proteins (4).

Hereditary diseases involving proteins of the TAL are classified into three categories according to the TAL function affected: sodium reabsorption, calcium and magnesium reabsorption, and uromodulin synthesis. The first category includes BS types 1–5 and HELIX syndrome (hypohidrosis, electrolyte imbalance, lacrimal gland dysfunction, ichthyosis, and xerostomia) (produced by mutations in *CLDN10* gene). The second category includes familial hypomagnesemia with hypercalciuria and nephrocalcinosis (produced by mutations in *CLDN16* or *CLDN19* genes), familial hypercalcemia hypocalciuric types 1–3 (produced by mutations in *CASR*, *GNA11*, *AP2S1* genes), and autosomal dominant hypocalcemia types 1–2 (produced by mutations in *CASR*, *GNA11* genes). Hyperuricemic nephropathy, familial juvenile 1 (produced by *UMOD* gene) belongs to the third category (4).

## Genes and Proteins in Bartter Syndrome

Bartter syndrome is caused by mutations in genes encoding K^+^ channel (*KCNJ1* gene), Cl^–^ channel (*CLCNKA* and *CLCNKB* genes), their cotransporters (*SLC12A1* gene), subunits of these channels (*BSND* gene), or regulators of the expression of certain transport channels (*MAGE-D2* gene).

**FIGURE 1:**
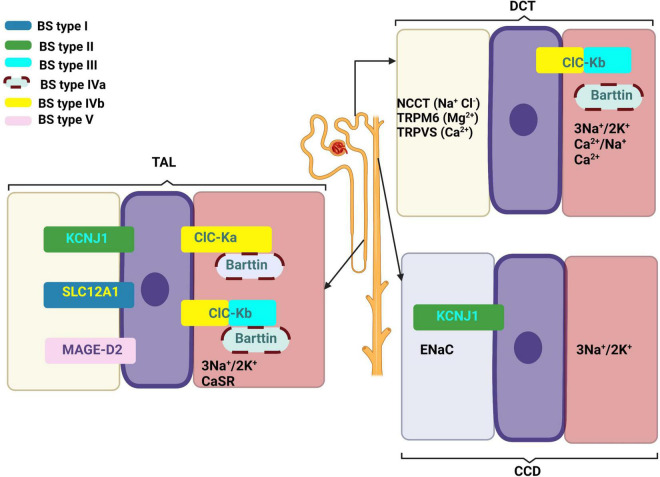
Gene expressions in different segments of the nephron and correspondent type of the Bartter syndrome. Created with BioRender.com.

Table 1 summarizes the genes implied in the pathogeny of various types of BS. Figure 1 describes the gene expressions in different segments of the nephron and correspondent type of the BS.

**TABLE 1:** Genes and proteins in Bartter syndrome (5–7).

Gene (Anterior/ alias symbol)	Approved name	Chromosomal location	Protein—Recommended name (Anterior/alternative name)	Bartter syndrome type
***SLC12A1*** *(NKCC2)*	Solute carrier family 12 member 1	15q21.1	**Solute carrier family 12 member 1** **(**Bumetanide-sensitive sodium-(potassium)-chloride cotransporter 2; Kidney-specific Na-K-Cl symporter**)**	**Type 1**
***KCNJ1*** *(Kir1.1* *ROMK1)*	Potassium inwardly rectifying channel subfamily J member 1	11q24.3	**ATP-sensitive inward rectifier potassium channel 1** **(**ATP-regulated potassium channel ROM-Kş Inward rectifier K (+) channel Kir1.1; Potassium channel, inwardly rectifying subfamily J member 1**)**	**Type 2**
***CLCNKB*** *(hClC-Kb)*	Chloride voltage-gated channel Kb	1p36.13	**Chloride channel protein ClC-Kb** **(**ClC-K2**)**	**Type 3**
***BSND*** ***(*** *BART* *DFNB73* ***)***	Barttin CLCNK type accessory subunit beta	1p32.3	**Barttin**	**Type 4a**
***CLCNKA*** *(hClC-Ka)*	Chloride voltage-gated channel Ka	1p36.13	**Chloride channel protein ClC-Ka** **(**ClC-K1**)**	**Type 4b**
***CLCNKB*** *(*hClC-Kb*)*	Chloride voltage-gated channel Kb	1p36.13	**Chloride channel protein ClC-Kb** **(**ClC-K2**)**	**Type 4b**
***MAGE-D2*** *(JCL-1, BCG1, 11B6, MAGE-D2, HCA10, MAGED, MGC8386)*	MAGE family member D2	Xp11.21	**Melanoma-associated antigen D2** **(**11B6, Breast cancer-associated gene 1 protein, Hepatocellular carcinoma-associated protein JCL-1, MAGE-D2 antigen**)**	**Type 5** **(Transient BS)**
***CASR***	Calcium sensing receptor	3q13.33-q21.1	**Extracellular calcium-sensing receptor**	**AD hypocalcemic hypercalciuria**

## Pathophysiology

**FIGURE 2:**
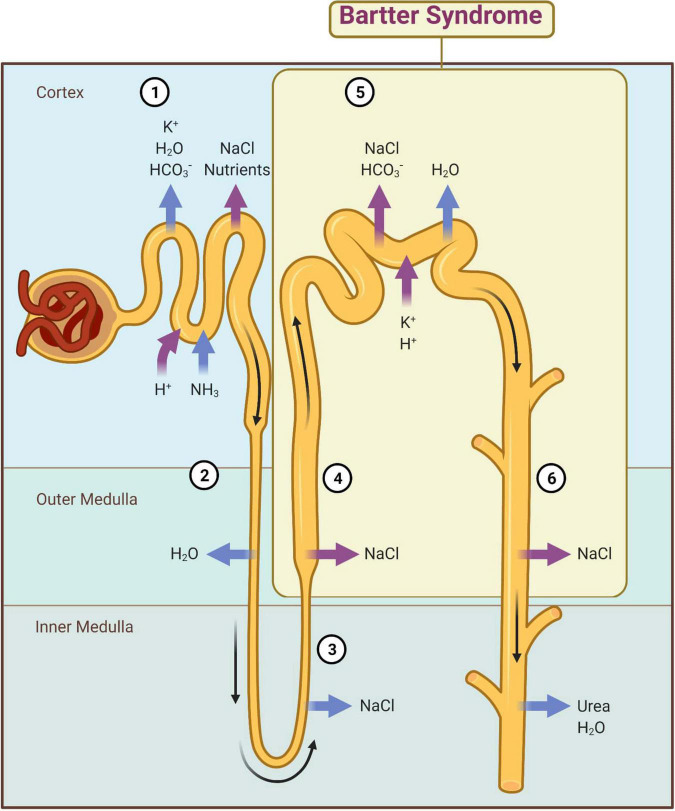
Main mechanisms of kidney reabsorption and secretion. 1—Proximal convoluted tubule; 2—Descending limb of loop of Henle; 3—Thin segment of ascending limb; 4—Thick segment of ascending limb; 5—Distal convoluted tubule; 6—Collecting duct; magenta arrow—active transport; blue arrow—passive transport. Adapted from “Kidney Reabsorption and Secretion,” by BioRender.com (2022). Retrieved from: https://app.biorender.com/biorender-templates (accessed on 22 March 2022).

At the descending limb of the loop of Henle, the urine becomes more and more hypertonic because this segment is permeable to water that passes extraluminal (passive transport) (8). At the level of the thin segment of the ascending limb, NaCl is reabsorbed according to the laws of osmosis. At the thick segment of the ascending limb and distal convoluted tubules (initial segment), Cl^–^, Na^+^, and K^+^ are actively reabsorbed (Figure 2). The result is a hypotonic urine. The collecting duct level is permeable to urea and water, and finally urine becomes hypertonic. These final changes at the nephron level are controlled by the antidiuretic hormone (ADH) that acts on the terminal segment of the distal tube and on the collecting duct (1). An important role in its regulation is played by the juxtaglomerular apparatus (JGA) which mediates the tubuloglomerular feedback (TGF). Under normal conditions, a decrease of intracellular concentration of Cl^–^ in JGA cells indicates a reduced filtration which determines an activation of TGF, with the stimulation of renin synthesis and hyperfiltration. In patients with BS, TGF is uncoupled because Cl^–^ is not reabsorbed into the macula densa. The cyclooxygenases are activated and large amounts of prostaglandin E2 are synthesized regardless of volume status, and this produces an excess of renin and aldosterone (1, 9).

Hypovolemia activates the renin-aldosterone system, with elevation of renin and aldosterone levels in blood and urine (10). The effects of aldosterone on the distal tubule are as follows: the increase of Na^+^ reabsorption from the lumen in exchange with K^+^ and the stimulation of exchange between the intracellular H^+^ and the intraluminal K^+^. In the first case, Na^+^/K^+^-ATPase pumps increase the intracellular K^+^ which results in an increase in the gradient with respect to the lumen. In this way, K^+^ will leave the cell in the lumen and in the urine (10). In the second case, metabolic alkalosis increases due to the loss of H^+^ in urine (10).

Thick ascending limb cells are responsible for 25–30% of the reabsorbed NaCl in the kidneys (4, 11).

The segments of the Henle loop (proximal straight tubule, thin descending, thin ascending limb, and TAL) are differentiated by the thickness of the walls and the properties of the epithelium. The descending and ascending segments are placed in the renal medulla, while the TAL is located distally. In the kidney medulla, the Na^+^/K^+^-ATPase has low levels and low transport activity. There are also differences between different cells and segments in terms of Na^+^/K^+^-ATPase activity, SLC12A1 splicing, and phosphorylation (11).

## SLC12A1

SLC12A1 is the major renal sodium, potassium, and chloride ion cotransporter expressed on the luminal membrane of renal epithelial cells of the TAL and macula densa. Its physiological functions are urine concentration, regulation of Ca^2+^ excretion, and reabsorption of NaCl in TAL (12). It is responsible for most of the NaCl absorption (6, 7).

All 12 solute carrier family proteins have some common properties: transport-coupled one cation per each transported anion (in the case of SLC12A1, for each one K^+^ and one Na^+^ that leave the cell enter two Cl^–^) which determines an electric balance; the anion is always the chlorine ion; their activity is modified by the changes in the cell volume; and the intracellular concentration of the chlorine ion; activity regulation is done by phosphorylation and dephosphorylation (12). It is positively regulated by WNK3 serine/threonine-protein kinase that activates SLC12A1 by phosphorylation and inhibits by dephosphorylation (12). This action is mediated by cation-chloride cotransporters (CCCs) dependent on cell tonicity (13). A decreased cell volume leads to phosphorylation of cotransporters by Serine/threonine-protein kinase STE20 (12, 14, 15).

Three full-length SLC12A1 isoforms exist in humans: isoform A, isoform B, and isoform F. Isoform A is the most intensely expressed (16, 17). However, there are differences in gene expression between different segments of TAL: isoforms A and F are mostly expressed in the outer stripe of the outer medulla, while isoform B is active in the cortex (16, 17). In addition, the affinity for Cl^–^ of isoforms decreases in this order: isoform B, isoform F, and isoform A, a feature concordant with spatial localization of these isoforms. Thus, the isoforms with higher affinity are expressed in C-TAL where there is a higher concentration of Cl^–^, while in M-TAL, the lower-affinity isoforms are present and concordant with low levels of Cl^–^ (16, 17).

## CIC-K

ClC-Ka and ClC-Kb are expressed differently in the epithelial cells of the nephron, inner ear, and salivary glands (18). The ClC-K channels have the largest protopore conductance values. These values range from 15 to 22 ps (18).

The ClC-K channels inserted in the basolateral membrane of nephron epithelia require a barttin subunit for proper function. Lack of barttin causes BS type 4a with renal loss of NaCl (7). ClC-Kb inactivating mutations cause BS type 3 (7, 17). ClC-Ka is expressed mainly in the thin limb of the Henle loop, while ClC-Kb is expressed in the TAL, distal-convoluted tubules, and in collecting duct-intercalated cells (7, 19). ClC-Ka, ClC-Kb, and barttin are also expressed in the ear where they are involved in the endolymph secretion. Both modified isoforms or their common beta subunit (barttin) cause deafness in BS type 4 (18).

## CLCNKA and CLCNKB

*CLCNKA* and *CLCNKB* are neighboring genes, present similar sequences, and probably result from the duplication of an ancestral gene. Deletions/non-sense mutations in *CLCNKB* produce loss of channel function which affects the response of the ClC-Kb complex to pH and Ca^2+^.

## ROM-K

ROM-K is an ATP-sensitive channel with low conductance, located at the apical pole of the cells. There is no difference between the expression ROM-K in M-TAL and C-TAL; it is expressed from the inner stripe of the outer medulla to the macula densa (17).

## MAGE-D2

MAGE-D2 is a nucleolar protein that increases *SLC12A1* expression in the human TAL and in the distal convoluted tubules. MAGE-D2 interacts with HSP40 allowing the protection of proteins. Also, MAGE-D2 in association with Gs-alpha promotes the insertion of SLC12A1 and NCC (NaCl-co-transporter) into the plasma membrane. In Barter type 5, due to the existence of an abnormal MAGE-D2, protein degradation is amplified, associated with low levels of SLC12A1 and NCC (4, 20).

## Clinical Signs

The typical symptoms in children with BS are polyhydramnios, premature delivery, polyuria, polydipsia, signs of hypovolemia, failure to thrive, and poor growth (21).

The age of presentation is different according to the type of mutation. For the BS types 4 and 5, polyhydramnios is earlier compared to BS types 1 and 2. The onset of BS type 3 is later in life and only rarely in the antenatal period (22).

There are rare cases of asymptomatic children diagnosed with BS. These cases presented hypokalemia metabolic alkalosis, normal blood pressure, and nephrocalcinosis, and usually such patients are discovered after a screening applied in a family with positive history of BS (23–26).

### Antenatal Symptomatology

The most frequent feature is the polyhydramnios detected during pregnancy, starting at 22 weeks of gestation (3). The onset of polyhydramnios is earlier in fetuses with BS types 4 and 5 (*BSND* or *MAGED2* variants) and represent the most severe forms. In BS type 1 (*SLC12A1*) and BS type 2 (*KCNJ1*), the onset of polyhydramnios is much tardive, concordant with a lower severity. In BS type 3 (*CLCNKB*), polyhydramnios is either absent or mild (3, 20).

Antenatal genetic testing and biochemical analysis of amniotic fluid, the concentration of total protein and alpha-fetoprotein (decreased in BS), the Bartter index (which is defined corresponding to the multiplication of total protein and of AFP, both expressed in multiple of median—MoM) with 86% sensitivity and 84% specificity, can be used to confirm the diagnosis (27, 28).

### Neonatal Symptomatology

All BS types, except BS type 3, have a neonatal onset with preterm birth (median gestational age between 29 and 33 weeks) and massive polyuria which lead to dehydration and rapid weight loss. Infants with BS type 4 have supplementary sensorineural hearing loss (22, 29).

The diagnosis can be confirmed using a next-generation sequencing (NGS) testing with gene panels that contain *SLC12A1*, *KCNJ1*, *BSND*, CLCNKA, *CLCNKB*, *MAGED2*, and *SLC12A3* gene. These tests have 75% sensitivity and 90–100% specificity (30).

### Childhood Symptomatology

Older infants and young children with BS type 3 fail to thrive, growth retardation, and present polyuria/polydipsia that lead to hypovolemia, persistent thirst, salt craving, constipation, unexplained fever, hypotonia, and recurrent vomiting (23–25).

### Teenage Symptomatology

Older children and adolescents with BS type 3 can present thirst, salt craving, fatigue, muscle weakness, cramps, nocturia, constipation, poor growth, and pubertal delay.

In BS type 4, there is a risk for chronic kidney disease (CKD) and end stage renal disease (ESRD) (22).

Bartter syndrome type 5 (transient type) has some extrarenal clinical features: frontal bone cyst, dysmorphic facies, hydrocephalus and Chiari malformation, Marfanoid habitus with arachnodactyly and mitral insufficiency, pyloric stenosis, high blood pressure, interauricular communication, left ventricle hypertrophy, right aortic arch, retroesophageal left subclavian artery, moderate pulmonary stenosis, enteropathic acrodermatitis zinc deficiency type, angioma, thrombocytopenia, and deafness after the age of 2 (3, 4, 31).

The laboratory findings constant in all forms of BS are hypochloremic metabolic alkalosis, elevated renin and aldosterone levels, low to normal blood pressure due to chronic hypovolemia, low urine osmolality due to impaired concentrating ability, and hypokalemia (potassium levels that are less than 3 mmol/L). Transient neonatal hyperkalemia may be present only in patients with BS type 2. Hypercalciuria is present in patients with BS types 1 and 2 (associated with nephrocalcinosis) and also in BS type 5 (the nephrocalcinosis is rare and mild in this type). The normocalciuria is present in patients with BS types 3 and 4, and the hypocalciuria in patients with BS type 3. The mild hypomagnesemia may be present in some patients with BS type 3. Plasma Cl/Na ratio can be normal in BS types 1 and 2, decreased in BS types 3 and 4, and increased in BS type 5 (1, 21, 32–35).

## Genetic Testing

Genetic testing is recommended in any clinical suspicion of BS. The European Rare Kidney Disease Reference Network Working Group for Tubular Disorders recommends a gene panel testing. This panel must contain not only all genes that cause BS and Gitelman syndrome but also genes that overlap phenotypically with BS and should be considered in differential diagnosis with BS: *SLC12A1, KCNJ1, CLCNKB, CLCNKA, BSND, MAGED2, SLC12A3, CASR, KCNJ10, SLC26A3, CLDN10, SCNN1A, SCNN1B, SCNN1G, NR3C2, HSD11B2, CYP11B1, CLCN2, KCNJ5*, and *CACNA1H* (1). Genetic testing is required for several reasons: confirmation of clinical diagnosis, adequate genetic counseling, adequate management of diseases with overlapping phenotypes, screening for deafness in BS type 4, and avoidance of aggressive treatment in transient BS type 5 (1).

**FIGURE 3:**
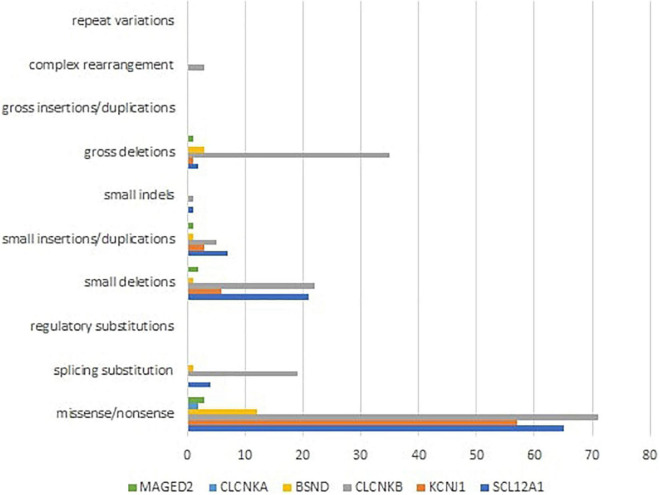
Different types of pathogenic variants in genes implied in Bartter syndrome (36).

Mutational heterogeneity is important in BS. According to the Human Gene Mutation Database Professional HGMD Professional 2021.4 (accessed in February 2022), 350 pathogenic variants were described. Figure 3 shows the implication in BS of different types of genetic modifications. The most common was missense/non-sense mutation, splicing substitution, small deletions, and gross deletion especially in *SLC12A1*, *CLCNKB*, and *KCNJ1* genes.

## Differential Diagnosis

The differential diagnosis of BS should be made with conditions where the main signs are poyhydramnios, salt loss, salt loss with hypokalemic alkalosis, hypokalemic alkalosis without salt loss, and nephrocalcinosis (1, 22).

The polyhydramnios may be a sign present in aneuploidia (the karyotype is abnormal), different gastrointestinal malformations, and congenital chloride diarrhea (dilated intestinal loops present).

The salt loss can be a manifestation in pseudohypoaldosteronism type I, but in this case is associated with metabolic acidosis and hyperkalemia.

The salt loss with hypokalemic alkalosis can be a sign in congenital chloride diarrhea, pseudo-BS (in cystic fibrosis, for example), surreptitious vomiting, surreptitious laxative use (in these entities, the additional finding is low urinary chloride), Gitelman syndrome (associated hypocalciuria and hypomagnesemia), HNF1B (hepatocyte nuclear factor 1 beta), nephropathy (associates renal malformation, MODY5—maturity onset diabetes of the young type 5, hypomagnesemia), HELIX syndrome (hypercalcemia is present), autosomal dominant hypocalcemia, EAST/SeSAME syndrome (seizures, sensorineural deafness, ataxia, mental retardation, and electrolyte imbalance), and surreptitious diuretic use (1, 22).

Hypokalemic alkalosis without salt loss associated hypertension with low renin/aldosterone and can be found in primary hyperaldosteronism, apparent mineralocorticoid excess, and Liddle syndrome (1, 22).

The nephrocalcinosis can also be found in distal renal tubular acidosis (metabolic acidosis), medical conditions without metabolic alkalosis (proximal tubular defects, familial hypomagnesemia/hypercalciuria), and apparent mineralocorticoid excess (1, 22).

## Therapy of Bartter Syndrome

The antenatal therapy consists of repeated amniocentesis and/or maternal administration of NSAID (non-steroidal anti-inflammatory drug) in order to reduce the amniotic fluid volume. There are potential risks for the fetus (such as necrotizing enterocolitis or premature closure of the ductus arteriosus). The evidence is not sufficient to show that the benefit outweighs the potential adverse effects (1). The main therapeutic interventions are summarized in Table 2.

**TABLE 2:** Treatment in Bartter syndrome (1, 40).

Therapeutic intervention	Doses	Evidence quality, strength of recommendation *	Comments
Na Cl supplementation	5–10 mEq/kg/d (mmol/kg/d)	Grade C (moderate recommendation)	Should be avoided in patients with BS types 1 and 2 who have secondary nephrogenic diabetes insipidus
KCl supplementation	2 mEq/d (mmol/kg/d)	Grade C (moderate recommendation)	The goal of therapy is to achieve a target of ≥ 3 mEq/L (mmol/L),
NSAID	Indomethacin (1–4 mg/kg/d divided in 3–4 doses) ibuprofen (15–30 mg/kg daily in 3 doses) celecoxib (2–10 mg/kg/d in 2 doses)	Grade B (moderate recommendation)	Should be accompanied by gastric acid suppression (for non-selective Cox inhibitors)
Nutrition Maximal caloric intake		Grade D (weak recommendation)	
Mg supplementation	oral organic magnesium salts (aspartate, citrate, lactate) 5 mg/kg (0.2 mmol/kg)	Grade D (weak recommendation)	The goal of therapy is to achieve a target of 1.46 mg/dL (0.6 mmol/L).
Potassium-sparing diuretics, angiotensin-converting enzyme inhibitors, and angiotensin receptor blockers	Diuretics (Spironolactone 1 mg/kg daily bid; Eplerenone 50 mg daily; Amiloride 10 mg daily); titrated to high doses Angiotensin-converting enzyme inhibitors (captopril 0.3–0.5 mg/kc bid-tid; enalapril 0.08–0.6 mg/kc qd; lizinopril 0.08–0.6 mg/kc qd) Angiotensin receptor blockers (candesartan 0.16–0.5 mg/kc qd; irbesartan 75–150 mg qd)	Grade D (weak recommendation)	May exacerbate renal salt wasting and increased polyuria

*Grade (B/C/D)—Evidence Quality; bid, bis in die (twice daily); tid, ter in die (three times a day); qd, quaque die (one a day).

The postnatal therapy consists of maximal caloric intake in order to facilitate optimal growth (1), fluid repletion, sodium chloride (NaCl) supplementation of at least 5–10 mEq/kg/d, and potassium chloride (KCl) 1–2 mEq/d, spread out in frequent doses throughout the day, in order to compensate the urinary losses (1, 37).

Non-steroidal anti-inflammatory drugs that inhibit prostaglandin E2, which contributes to high urinary NaCl losses, are recommended in case of inadequate response in symptomatic patients with BS, especially in early childhood (38). Oral magnesium supplementation is recommended in case of hypomagnesemia [<1.70 mg/dl (0.7 mmol/L)] (37, 39). In case of symptomatic hypokalemia despite the supplements, potassium-sparing diuretics (such as spironolactone, eplerenone, or amiloride), angiotensin-converting enzyme inhibitors, and angiotensin receptor blockers can be used (1, 37).

## Follow-Up in Bartter Syndrome

The patients with BS should be followed in pediatric or adult centers experienced in tubular disorders. The recommended frequency of hospital visits is 3–6 months for infants and young children and 6–12 months for adults. All the recommendations are summarized in Table 3. A clinical work on hydration status, degree of polyuria, muscular weakness, growth, and psychomotor development is recommended at each follow-up visit (1, 22).

**TABLE 3:** Follow-up in Bartter syndrome (1, 22).

	Frequency of visits in centers	Clinical work up	Biochemical work up	Cardiac work up	Renal ultrasound
Infants	3–6 months	At each follow up visit	At each follow up visit		12–24 months
Young children	3–6 months	At each follow up visit	At each follow up visit		12–24 months
Older children	6–12 months	At each follow up visit	At each follow up visit		12–24 months
Adult patients	6–12 months	At each follow up visit	At each follow up visit	In case of palpitations or syncope	12–24 months
Level of recommendation	Grade C	Grade C	Grade C	Grade C	Grade C

A biochemical work focused on metabolic acid-base status (either blood gas or by measurement of venous total CO_2_), serum electrolytes (natremia, potassium, chloride, magnesium, and bicarbonate levels), renal function, microalbuminuria, urinary calcium excretion, PTH, and urine osmolality for the detection of nephrogenic diabetes insipidus is recommended at each follow-up visit (1, 22).

Growth hormone deficiency may be considered for children with growth retardation despite treatment.

Renal ultrasound is recommended to be performed at least every 12–24 months to monitor the occurrence of kidney stones, nephrocalcinosis, and obstructive uropathy.

Cardiac work out is recommended for patients (particularly adults) with palpitations or syncope. The physician can use the drugs that slow the sinus rhythm (beta-blockers, calcium channel blockers as verapamil, diltiazem, digoxin) or drugs that influence the QT interval (proton pump inhibitors, fluoroquinolones, macrolides, gentamicin, or antiviral drugs). Electrocardiography, Holter, and stress electrocardiography are recommended to detect cardiac arrhythmias which are determined by a prolonged QT interval in the context of hypokalemia and hypomagnesemia (1, 22).

The quality of life is important to be assessed by using age-specific standardized questionnaires. The quality-of-life scores are related by biochemical parameters (potassium and aldosterone) (1, 41).

Sports can be performed with a good hydration and additional salt and electrolytes.

Anesthesia for patients with BS must be performed after improvement of hypokalemia and hypomagnesemia, which in addition with anesthetic agents can lead to neuromuscular blockage. Potassium levels > 3.0 mmol/L and magnesium > 0.5 mmol/are suggested by guidelines (42).

### Kidney Disease

Renal disease is characterized by urolithiasis, obstructive uropathy, and nephrocalcinosis. Nephrotic-range proteinuria has also been reported in patients with BS (21, 24, 43). Renal biopsy can show diffuse glomerular and tubule-interstitial lesions with enlarged glomeruli and focal segmental glomerulosclerosis (23).

Only some patients have an evolution to end-stage kidney disease. CKD can occur as a late manifestation in patients with BS, particularly types 1, 4a, and 4b (21, 23, 44). The CKD in BS seems to have several mechanisms: chronic stimulation of the renin–angiotensin system, periodic dehydration, prematurity, nephrocalcinosis, long-term NSAID drug treatment, and chronic hypokalemia (4).

There are few reported cases of kidney transplants (23, 45–50). The electrolytes and urinary concentrating abnormalities were corrected. No evidence of recurrent disease was observed.

Few studies reported growth failure with growth hormone (GH) deficiency in patients with BS, particularly type 3 who have severe metabolic abnormalities. The poor growth explanation is unclear: acid-base or electrolyte disturbances in BS or whether it is an intrinsic part of the disorder. After metabolic control, recombinant human GH supplementation can be performed (21, 24, 51–53).

## Conclusion

Knowledge of clinical heterogeneity in BS is important for initiating genetic investigations and proper management. Early molecular diagnosis is desirable for personalized therapy.

## Data Availability Statement

Some data was obtained from Human Gene Mutation Database (HGMD^®^ Professional 2021.4) available online: http://www.hgmd.cf.ac.uk/ac/all.php (accessed February 15, 2022).

## Author Contributions

LF, LC, and EG: writing – original draft, review and editing. All authors contributed to the article and approved the submitted version.

## Conflict of Interest

The authors declare that the research was conducted in the absence of any commercial or financial relationships that could be construed as a potential conflict of interest.

## Publisher’s Note

All claims expressed in this article are solely those of the authors and do not necessarily represent those of their affiliated organizations, or those of the publisher, the editors and the reviewers. Any product that may be evaluated in this article, or claim that may be made by its manufacturer, is not guaranteed or endorsed by the publisher.

## Abbreviations

CLDN10: claudin 10
CLDN16: claudin 16
CLDN19: claudin 19
CASR: calcium sensing receptor
AP2S1: adaptor related protein complex 2 subunit sigma 1
GNA11: G protein subunit alpha 11
UMOD: uromodulin
SLC12A1: solute carrier family 12 member 1
KCNJ1: potassium inwardly rectifying channel subfamily J member 1
CLCNKB: chloride voltage-gated channel Kb
CLCNKA: chloride voltage-gated channel Ka
BSND: barttin CLCNK type accessory subunit beta
MAGED2: MAGE family member D2
SLC12A3: solute carrier family 12 member 3
CASR: calcium sensing receptor
KCNJ10: potassium inwardly rectifying channel subfamily J member 10
SLC26A3: solute carrier family 26 member 3
CLDN10: claudin 10
SCNN1A: sodium channel epithelial 1 subunit alpha
SCNN1B: sodium channel epithelial 1 subunit beta
SCNN1G: sodium channel epithelial 1 subunit gamma
NR3C2: nuclear receptor subfamily 3 group C member 2
HSD11B2: hydroxysteroid 11-beta dehydrogenase 2
CYP11B1: cytochrome P450 family 11 subfamily B member 1
CLCN2: chloride voltage-gated channel 2
KCNJ5: potassium inwardly rectifying channel subfamily J member 5
CACNA1H: calcium voltage-gated channel subunit alpha1 H.
